# Age-Appropriateness of Oral Formulations Authorized for Pediatrics

**DOI:** 10.1007/s11095-026-04055-x

**Published:** 2026-03-16

**Authors:** Hannah R. Weiler, Lauren M. Le, Hala M. Fadda

**Affiliations:** Department of Pharmaceutical Sciences, College of Pharmacy and Health Sciences, Butler University, Indianapolis, IN 46208 USA

**Keywords:** chewable tablets, essential medicines, modified release, orally disintegrating tablets, WHO

## Abstract

**Purpose:**

Pediatric global regulatory initiatives have come into place to address gaps in evidence-based medicine for children. Objective of this study was to assess age-appropriateness of oral pediatric formulations authorized through the U.S. Food and Drug Administration’s Pediatric Rule, Best Pharmaceuticals for Children Act and Pediatric Research Equity Act.

**Methods:**

Formulation age-appropriateness was assessed using an adapted version of the World Health Organization pediatric quality product profile assessment tool. Evaluations encompassed four product attributes across pediatric subgroups: dose and dose flexibility, patient acceptability, excipient safety and administration considerations. Each attribute was scored on a three-point scale: 1 indicating high risk, 2 moderate risk, and 3 low risk.

**Results:**

214 oral formulations were evaluated. Age-appropriateness remains a concern; with less than one third of authorized oral formulations for neonates meeting criteria for age-appropriateness, in comparison to 66% for older pediatric populations. Tablets and capsules constitute 58% of formulations indicated for pediatrics, despite tablet sizes often being unsuitable for younger children. Liquid formulations for neonates present a significant oversight, as they frequently contain excipients of potential concern. As children age, swallowability of the dosage form appears to be deprioritized, with only 37% of unswallowable large size tablets for school-aged children, available in alternative dosage forms such as granules, or solids for dispersions.

**Conclusions:**

Significant formulation gaps persist in pediatric medicine, with acceptability and excipient safety presenting highest risk. The potential of non-conventional solid dosage forms remains underutilized, underscoring the need for regulatory frameworks to support development of pediatric medicines.

**Supplementary Information:**

The online version contains supplementary material available at 10.1007/s11095-026-04055-x.

## Introduction

The off-label prescribing of medicines in pediatric populations remains a widespread global practice, with reports indicating rates as high as 70% prior to the implementation of international regulatory frameworks [1, 2]. This prevalence stems from longstanding ethical constraints surrounding the inclusion of children in clinical research, compounded by logistical complexities and limited commercial incentives for pharmaceutical companies to pursue pediatric-specific drug approvals. Risks associated with off-label prescribing include use of medicines that lack therapeutic efficacy, increased risk of adverse events, and dosing errors [3].

The United States (U.S.) Food and Drug Administration (FDA) and European Medicines Agency (EMA) have implemented regulations to improve pediatric drug development and increase the availability of safe and efficacious medicines for children. The FDA Best Pharmaceuticals for Children Act (BPCA) of 2002 provides an incentive of six months marketing exclusivity for voluntary conduction of agreed upon pediatric studies for new medicines [4]. The FDA Pediatric Research Equity Act (PREA) of 2003 [5] is a revision of the Pediatric Rule introduced in 1998 and later repealed in 2002 [6]. The original rule mandated that new active pharmaceutical ingredients (APIs) are not authorized unless sponsors include all available data on efficacy and safety in children. Alternatively, PREA authorizes regulatory bodies to require pediatric research in all relevant pediatric subpopulations for new drug products being developed, including new APIs, indications, dosage regimens, routes of administration, or dosage forms. In 2007, EMA mandated the European Union Pediatric Regulation (PR), requiring pediatric drug development for all new drug products and rewarding a 6-month supplementary protection certificate (SPC) that extends patent life [7].

Regulatory initiatives have significantly increased the availability of authorized pediatric medicines. Between 2007 and 2016, 260 new medicinal products were approved by EMA for pediatric use within the European Union [8]. The FDA authorized 797 APIs for pediatric use during the period from 1998 to 2024 [9]. Despite regulatory advancements, significant challenges persist in the development of pediatric medicines. Sponsors may request deferrals from the FDA and EMA, thereby extending the timeline for the completion of pediatric studies. Notably, only 33.8% of the pediatric post marketing studies mandated under PREA have been completed, with a median follow-up duration of 6.8 years [10]. Moreover, older, off-patent drugs continue to receive little attention due to their limited commercial viability. Pediatric drug development is further complicated by the inherent heterogeneity of the pediatric population. This demographic spans from preterm neonates to adolescents aged 16 or 18 years, depending on regional definitions, with body weights potentially exceeding 100 kg. Such variability necessitates flexible dosing strategies to ensure therapeutic efficacy and acceptability across age groups. Furthermore, conducting pharmacokinetic and bioequivalence studies across these diverse pediatric subpopulations presents challenges [1]. These complexities may explain why pediatric oral extemporaneous compounding continues to be a common global practice, with eight of the 20 most compounded oral pediatric medicines listed on the WHO Essential Medicines List for Children (EMLc) [11].

Sponsors developing new medicines, as well as those pursuing new indications, dosage forms, or routes of administration, are required to present age-appropriate formulations for the pediatric population. An age-appropriate medicine is defined as “a medicine whose pharmaceutical design should be suitable for use in the target age group(s)” [12]. An assessment of Pediatric Investigation Plans (PIPs) submitted to EMA found that 93% of the formulations reviewed raised at least one concern related to excipient safety, dose accuracy, patient acceptability, or the suitability of the dosage form or route of administration [13]. A separate investigation of the age-appropriateness of enteral formulations of medicines listed on the WHO EMLc between 2011 and 2019 found that the majority of these medicines were not age-appropriate based on swallowability and dose flexibility [14].

This study undertakes a comprehensive assessment of the age-appropriateness of oral pediatric medicines approved in the United States through the Pediatric Rule, PREA or BPCA pathways. To evaluate formulation age-appropriateness, an adapted version of the WHO pediatric quality product profile assessment tool (PQPPAT) was utilized [15]. The WHO PQPPAT is derived from the well-established quality target product profile (QTPP) utilized in pharmaceutical development which provides a prospective summary of the ideal quality characteristics of the drug product [16]. The WHO PQPPAT focuses on attributes pertinent to pediatric pharmaceutical products. This investigation represents the first systemic evaluation of the age-appropriateness of all oral formulations approved for children up to 12 years of age under U.S. pediatric regulatory frameworks. In addition to assessing formulation suitability, this study categorizes oral formulations by therapeutic area and examines the distribution of dosage form types across pediatric age groups. It also explores the presence of excipients with a potential concern in oral liquid formulations. Ultimately, this research seeks to identify gaps in current pediatric oral formulations to inform and support the development of safer and more acceptable pharmaceutical products for children.

## Methods

Oral pediatric medicines approved for children up to 12 years of age under the Pediatric Rule, PREA, and BPCA pathways from 1998 to mid-2024, were evaluated for age-appropriateness using the WHO PQPPAT. This study focuses on oral formulations, the predominant route for chronic disease management, where long-term adherence and patient acceptability are pivotal for therapeutic success. While parenteral administration presents its own complex challenges regarding age-appropriateness, it is reliant on specialized training or healthcare professionals, and therefore creates a distinct set of acceptability issues that require other standardized evaluation criteria. Drug formulations were identified for analysis via an FDA-maintained spreadsheet of all pediatric labeling changes made under the Pediatric Rule, BPCA, and PREA [9].

Considering the heterogeneity of the pediatric patient population, separate evaluations were conducted for each of the pediatric subgroups stratified by age: (i) term newborn infants, neonates (0 to 27 days); (ii) infants and toddlers (28 days to 23 months); (iii) preschool children (2 to 5 years); and (iv) school children (6 to 11 years). The age categories applied in this study were based on the pediatric clinical trial age classifications recommended by the U.S. National Institute of Child Health and Human Development and International Council for Harmonization [17, 18]. U.S. product labeling typically specifies pediatric indications using these age brackets, facilitating accurate characterization of our dataset. When dosing was based on weight, it was mapped to the average corresponding age for children [19]. The WHO PQPPAT evaluates seven attributes of pharmaceutical products, specifically: (i) target population (age); (ii) dose and dose flexibility; (iii) patient acceptability; (iv) excipient safety; (v) administration considerations; (vi) stability, storage conditions, and primary packaging materials; and (vii) registration status [20]. For the purpose of this study, the PQPPAT was adapted to focus on attributes two through five. Target population and registration status were not assessed, since separate evaluations were conducted for each age group and the current study investigated only authorized medicines. Stability, storage conditions, and primary packaging material were also excluded from evaluation, as all authorized oral formulations sufficiently meet these criteria. Only oral formulations approved for children up to 12 years of age were evaluated, aligning with the scope of the WHO PQPPAT. Products indicated for children older than 12 years were excluded; although clinically defined as pediatric, this age group shares similar medication acceptability profiles with adults.

Scoring criteria for each attribute evaluated in this study are provided in Table I. Acceptability scoring of solid dosage forms was determined based on established dimensional thresholds derived from observational studies of spontaneous swallowing ability in untrained populations. Recent evidence suggests that children as young as six months can swallow multiple tablets of 2 mm (mm) in size [21]. Moreover, oblong tablets with dimensions of 2.5 × 6 mm, were shown to have good acceptability, swallowability, and palatability in children between 1 −5 years of age [22]. School age children (6- 11 years) demonstrated the ability to swallow 15 mm tablets [23]. Furthermore, most school children could swallow larger tablets measuring 21 × 11 × 7 mm [24]. It important to note, however, that the threshold assessments utilized in our study do not account for enhanced swallowing capacity following systematic training interventions (e.g., ‘Pill School’ programs) [25]. Swallowing training programs are not uniformly available across healthcare settings and therefore our assessments reflect the real-world acceptability of oral formulations for pedaitrics.

**Table I:** Attribute scoring criteria

Target attribute	Low risk/no issues Partially meet target Score = 3	Moderate risk/Issues Partially meets target Score = 2	High risk/Issues Does not meet target Score = 1
Dose, dose flexibility	Available strengths allow accurate and precise administration of *all* pediatric doses	Available strengths allow accurate and precise administration of *some* pediatric doses	Available strengths *do not* allow accurate and precise administration of pediatric doses
Patient acceptability			
0–5 yrs	Liquid vol. ≤ 5 mL, multiparticulates	Liquid vol. ≤ 5 mL, tablets < 6 mm	Tablets ≥ 6 mm
6–12 yrs	Multiparticulates, tablets ≤ 15 mm, ODTs, chewables	Liquid vol. ≤ 10 mL. tablets 16–20 mm	Liquid vol. > 10 mL tablets ≥ 21 mm
Excipient Safety	*No* potentially harmful excipients	1 to 2 potentially harmful excipients	≥ 3 potentially harmful excipients *or* contains ethanol or propylene glycol if child < 6 yrs old
Administration conditions	*No* manipulation or measurement required	1 unideal administration requirement	≥ 2 unideal administration requirements (e.g. reconstitution with vehicle, complex dose measurement, use with specific diets or food items, or multiple daily doses)

Attributes and scores are adapted from WHO PQPPAT [15]

*ODT* orally disintegrating tablets; tablet size refers to tablet or capsule size

Multiple daily doses are considered unideal when administered three times daily with food or four times daily without food

Drug formulation evaluations with respect to the aforementioned target attributes were assessed individually utilizing information from the manufacturers’ labeling approved under the products’ original and supplemental New Drug Applications, as well as from accredited drug databases, including DailyMed and UpToDate LexiDrug [26, 27]. Final subscores for pediatric age-appropriateness were subject to adjustment based on characteristics not explicitly defined within the guidelines, such as palatability of the formulation per the presence or absence of flavoring. Each evaluation was reviewed by another team member to ensure consistency of assessments. For each age group, individual scores were aggregated to yield a total product score. Scores ranging from 10 to 12 were classified as age-appropriate, those between 7 and 9 as partially age-appropriate, and scores from 4 to 6 as not age-appropriate. APIs were classified according to the WHO Anatomical Therapeutic Chemical (ATC) classification system second level pharmacological or therapeutic subgroups [28].

## Results and Discussion

### Oral Formulations Authorized for Pediatrics

**Fig. 1:**
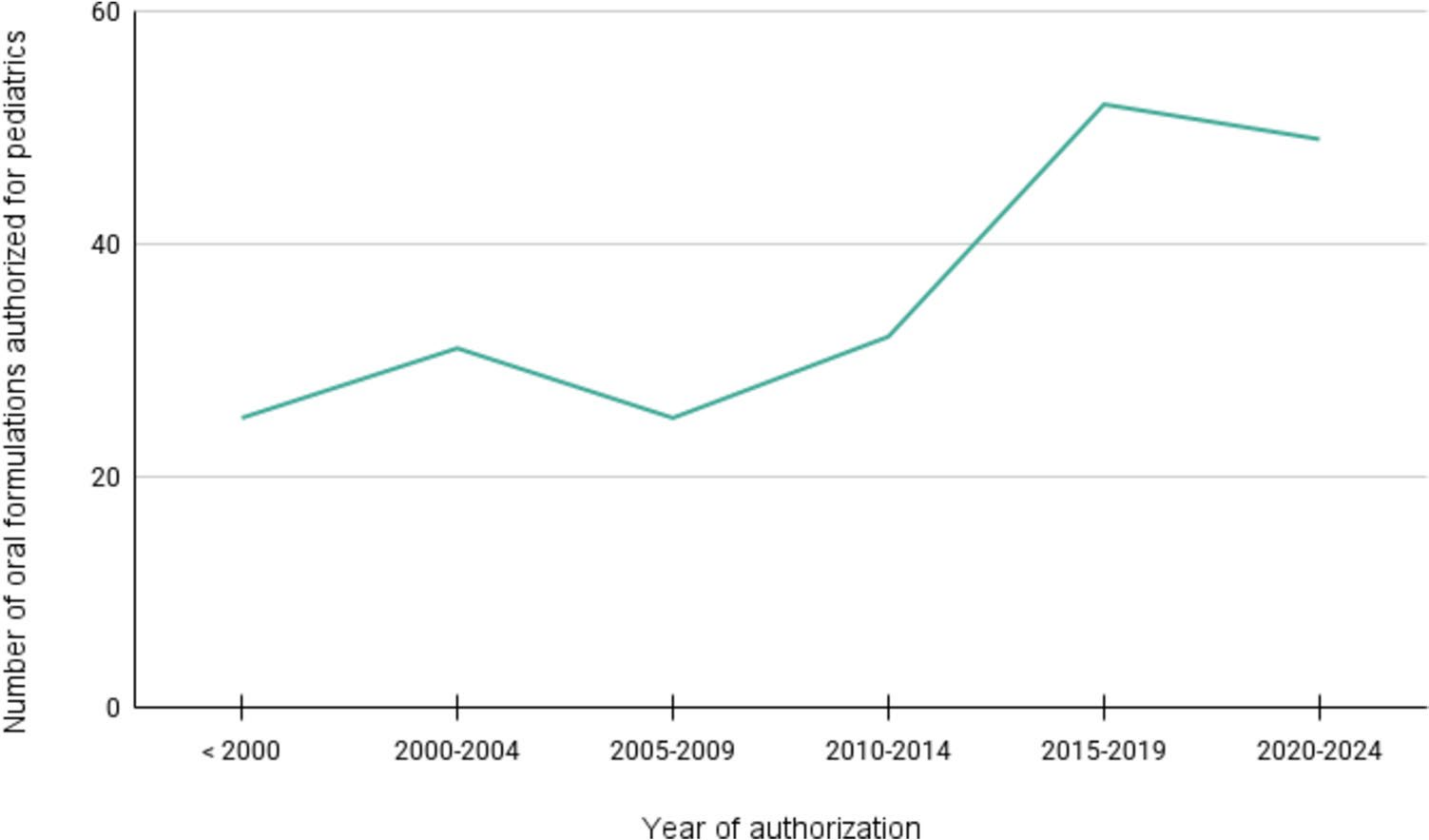
Number of oral formulations authorized for pediatrics through Pediatric Rule, PREA and BPCA pathways.

Between 2002 to 2024, 123 orally administered APIs received pediatric authorization for children up to 12 years of age, comprising 214 unique oral formulations (supplementary table). Figure 1 illustrates a significant increase in the number of authorized pediatric oral formulations from 2015 onwards. This may be attributed to the time taken to receive authorization following the submission of pediatric study plans, considering the challenges associated with conducting pediatric studies and deferrals requested by sponsors. It should also be emphasized that this investigation was limited to oral formulations that had received FDA approval as of mid-2024.

### Oral Formulations Authorized for Pediatrics per Therapeutic Class

**Fig. 2:**
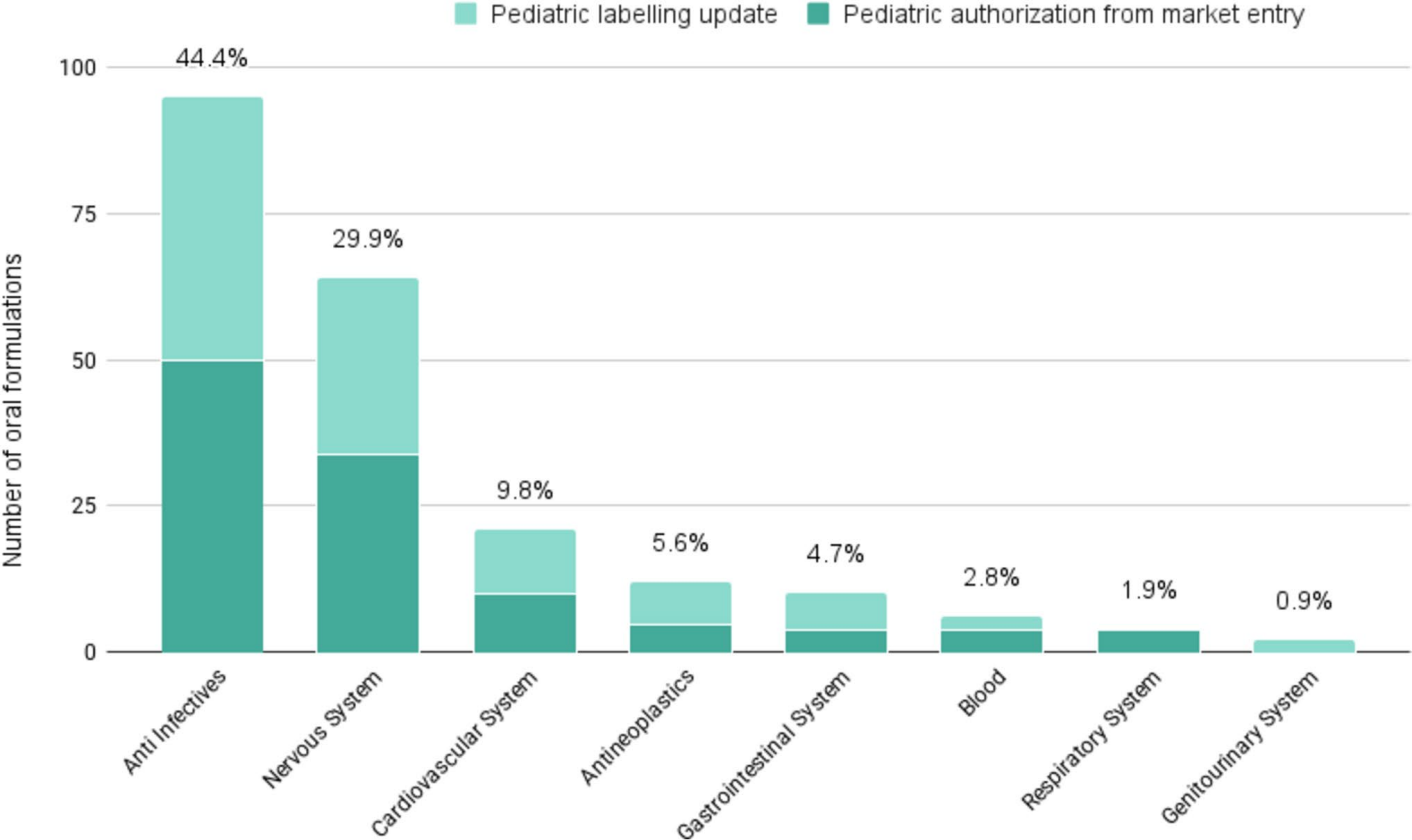
Pediatric authorizations of oral formulations at the time of market entry *vs* pediatric approvals post authorization for adults. Percentages indicate oral formulations authorized for pediatrics per therapeutic class. Note. Anti Infectives include one antiparasitic product and one pharmacokinetic enhancer used for HIV treatment.

According to the WHO ATC second level categorization, anti-infectives constitute the majority of oral formulations approved for pediatric use. This is followed by oral formulations targeting the nervous system and, subsequently, the cardiovascular system (Fig. 2). Within the nervous system category, anticonvulsants are the most authorized, followed by medicines indicated for attention-deficit hyperactivity disorder.

Of the 101 anti-infective oral formulations profiled, 71 are antiviral, 24 are antibacterial, and six are antifungal. Notably, 50 of the 71 antiviral formulations are indicated for the treatment of Human Immunodeficiency Virus (HIV). From the enactment of the Ryan White Comprehensive AIDS Resources Emergency (CARE) Act in 1990 to the sustained global efforts led by the U.S. President’s Emergency Plan for AIDS Relief (PEPFAR) in 2003, pediatric HIV drug development has benefited from an exceptional level of advocacy, funding coordination, and regulatory support [29, 30]. This convergence of public health urgency and international investment has positioned HIV as a model case for pediatric drug development. By contrast, many other disease areas lack the same alignment of incentives and resources, resulting in more limited progress in pediatric research.

111 oral formulations were approved for pediatric use at the time of initial market entry while 103 gained pediatric approval through labeling changes, highlighting the impact of BPCA and PREA (Fig. 2). For most therapeutic categories, 50% of the oral formulations were authorized for pediatric use after being authorized for adults. Sponsors may request to defer pediatric study plans until sufficient efficacy and safety data have been obtained in adults adults. This approach aims to protect pediatric populations while preventing delays in the development of adult therapeutics [31]. All four of the oral formulations for the respiratory system, however, were authorized for pediatrics at the time of market entry. These formulations, indicated for the treatment of cystic fibrosis, include ivacaftor granules and tablets, as well as fixed-dose combinations of lumacaftor and ivacaftor in both granule and tablet forms. In contrast, the two mirabegron formulations, tablets and granules for suspension, approved for the treatment of neurogenic detrusor overactivity within the genitourinary system, underwent pediatric labeling updates following market authorization for adults.

### Age-Appropriateness of Oral Formulations Authorized for Pediatrics

**Fig. 3:**
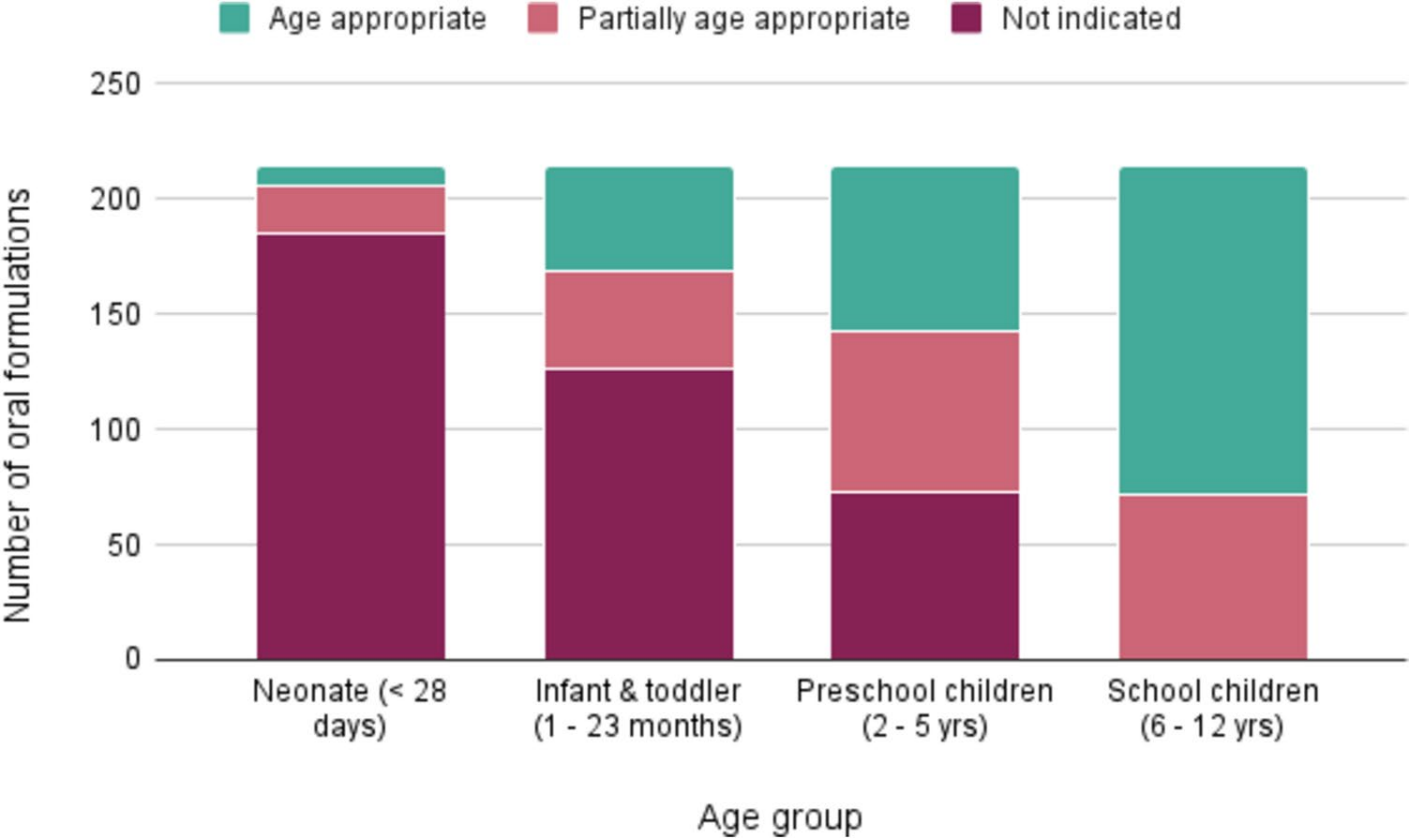
Age-appropriateness of oral formulations authorized for pediatrics.

The number of oral formulations authorized for pediatric use increases with pediatric age (Fig. 3). Most pediatric oral medicines approved through PREA and BPCA pathways are not indicated for neonates nor infants and toddlers. For neonates, infants and toddlers, preschool children, and school children, 28, 87, 140 and 214 oral formulations are authorized for pediatric use, respectively. Less than one third of authorized oral formulations for neonates are age-appropriate, compared to approximately 50% for infants and toddlers and pre-school children. Oral formulations authorized for use in older pediatric populations tend to achieve higher PQPPAT scores. This improvement in age-appropriateness may be attributed to the greater acceptability of oral dosage forms among older children, coupled with less stringent excipient safety requirements. Comparable results were reported in a 2011 study of medicines in the Netherlands, which found that a significant proportion of authorized pediatric medicines lacked age-appropriate formulations. The study highlighted that appropriateness, particularly with respect to dose flexibility and dosage form suitability, improved with increasing pediatric age, rising from 27% among neonates to 70% among school-aged children (6–11 years) [32].

**Fig. 4:**
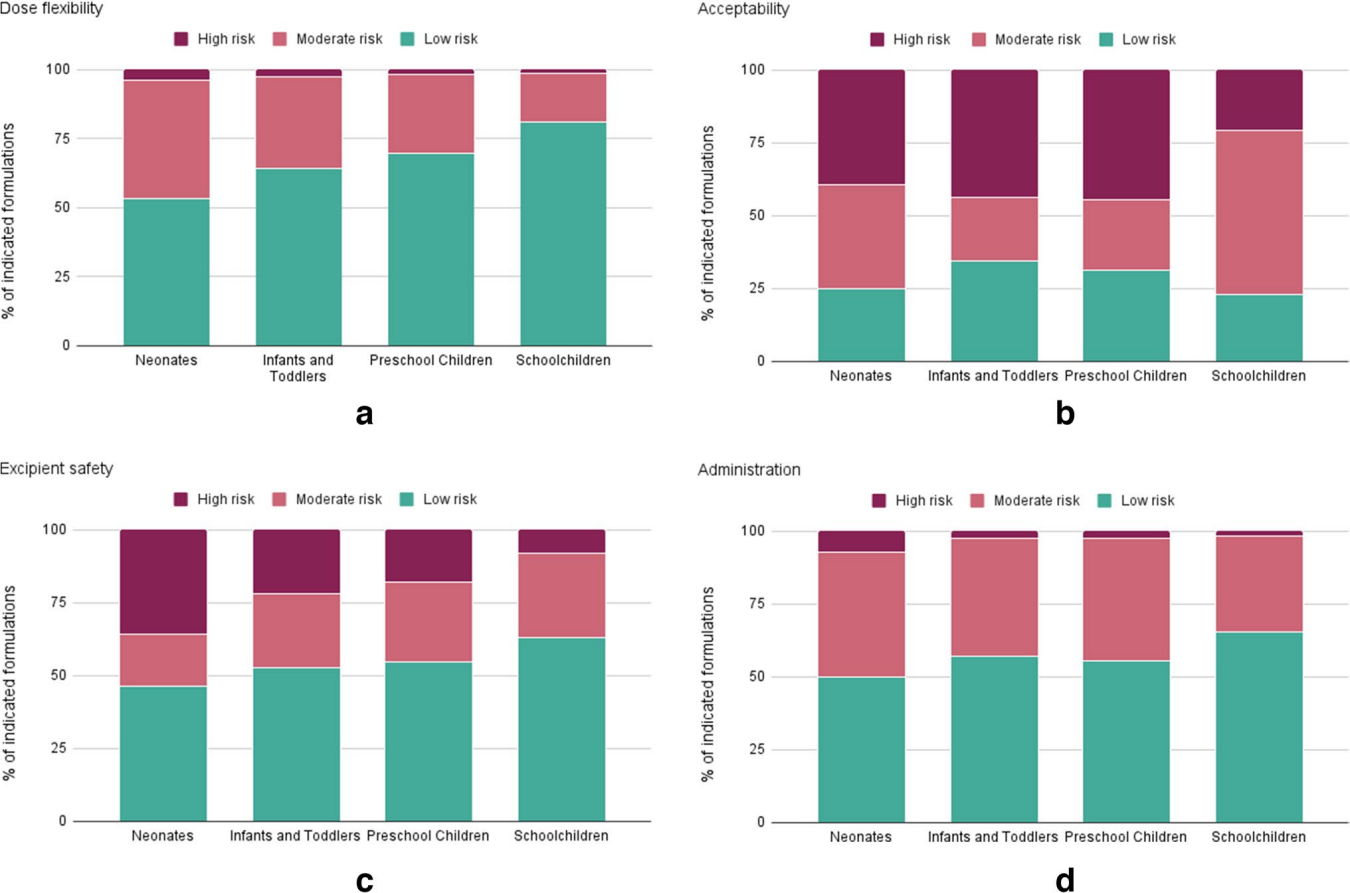
Risk associated with each product attribute for pediatric subgroups (**a**) Dose flexibility risk (**b)** Product acceptability risk (**c**)Excipient safety risk (**d**) Product administration risk.

The risk associated with each of the four evaluated product attributes was assessed for all pediatric age groups (Fig. 4). Notably, the risk related to dose flexibility demonstrated an age-dependent improvement, with moderate risk observed in 43% of oral formulations authorized for neonates, decreasing to 18% among those approved for school-aged children. Our findings demonstrate that accurate dose administration remains challenging for certain pediatric patients, particularly when unscored tablets require manipulation to achieve the intended dosage. This suggests that the available strengths may not consistently support precise and reliable dosing across all pediatric subpopulations. Nonetheless, fewer than 5% of the authorized oral formulations were associated with a high risk in any pediatric age group.

Acceptability presented a higher product profile risk, with approximately 40% of products demonstrating high risk in neonates, infants and toddlers, and preschool children. This elevated risk predominantly stems from the size of conventional tablets and capsules, which are often unsuitable for swallowing in these age groups. Additionally, volumes of liquid formulations exceeding recommended thresholds further contribute to acceptability challenges.

Excipient safety presented the highest risk for neonates, with 36% of formulations displaying high risk. This can be attributed to the higher prevalence of liquid preparations authorized for neonates given their inherent inability to swallow solid dosage forms. Liquid formulations containing ethanol or propylene glycol were categorized as high risk for neonates, and infants and toddlers, since the enzyme responsible for metabolism of these two cosolvents, alcohol dehydrogenase, does not fully mature until 5 years of age [33].

Administration presented a moderate risk for approximately 40% of the formulations authorized for neonates, infants and toddlers, and preschool children. Moderate-risk products often require manipulation, complex measurement, or unideal administration methods, such as reconstitution with a specific vehicle. Administration was determined to be at high risk for approximately 7% of formulations authorized for neonates and approximately 2% for all other pediatric age groups.

Table II illustrates the nuanced interplay between perceived and actual barriers to achieving age-appropriate oral formulations in pediatric drug development. Swallowability emerges as a widely acknowledged determinant of age appropriateness, particularly for younger subpopulations, as evidenced by the availability of swallowable alternatives for 100% and 89% of indicated products deemed unswallowable by neonates, and by infants and toddlers, respectively. However, the emphasis on liquid formulations for neonates, infants and toddlers, presents a critical oversight: excipients of potential concern present in 80% and 50% of liquid alternatives for neonates, and for infants and toddlers, respectively. Conversely, swallowability appears to be unjustly deprioritized as children age, with our findings indicating that only 37% of unswallowable products for school-aged children are available in alternative acceptable alternatives such as liquids, granules or solids for dispersion. Overall, the study suggests that the drivers of partial age appropriateness in oral pediatric products vary by age group, with specific formulation attributes posing differential risks across developmental stages.

**Table II:** Non- swallowable oral solid formulations and alternative acceptable formulations

	Non-swallowable solid formulations	Alternative acceptable (solid/liquid) formulations	Alternative liquid formulations with excipients of potential or known concerns
Neonates	10	10	8
Infants and Toddlers	36	32	16
Preschool Children	60	48	18
School children	30	11	0

Criteria outlined in Table I were used to evaluate the swallowability of oral solid formulations and to determine formulations with high-risk excipients

### Distribution of Oral Formulations of Authorized Pediatric Medicines

**Fig. 5:**
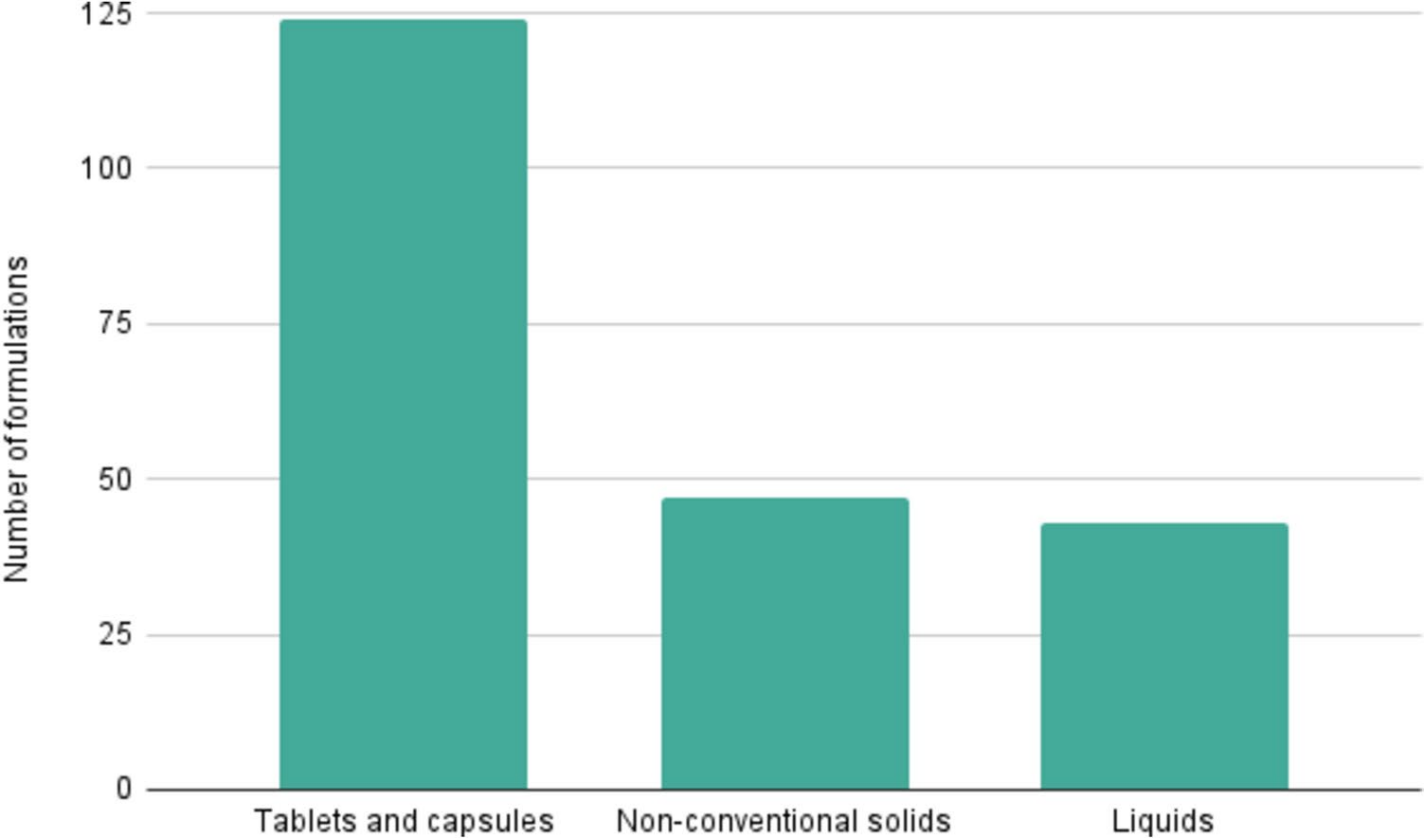
Distribution of oral formulations authorized for pediatrics.

Formulations of authorized oral pediatric medicines ranged from liquids to multiparticulates to tablets up to 22 mm in diameter. Figure 5 illustrates the distribution of dosage form types. Solutions, suspensions, and syrups were classified as liquids, and these comprised 20% of approved dosage forms authorized for pediatrics. Interestingly, however, tablets and capsules comprised most dosage forms at 58%. The remaining 22% of formulations were classified as ‘non-conventional solids’; these include sublinguals, orally disintegrating tablets (ODTs), chewable tablets, as well as multiparticulate systems encompassing powders, mini-tablets (granules), pellets, pellet/tablet-filled capsules, and powders/granules for suspension. Mini-tablets have shown promise with respect to swallowability and acceptability in children, with potential for successful scale-up and film coating [34, 35].

It is noteworthy that even with the presence of advanced oral formulations, including ODTs and modified release multi-particulate ion-exchange resins, single-unit tablets and capsules still predominate the authorized oral medicines market for pediatrics. This is especially compelling considering that PREA and PR require the use of age-appropriate formulations for each subset of the pediatric population they will be tested in [12, 36]. While PREA requires sponsors to develop age-appropriate formulations to conduct pediatric studies, it does not require the formulations to be marketed.

In 2017, the FDA approved Spritam® (levitracetam 250, 500, 750, 1000 mg) (Aprecia, OH, USA), the first 3D printed ODT indicated for epilepsy in children from 4 years of age. Spritam is manufactured by drop on powder 3D printing, thus creating a porous tablet that rapidly disintegrates with a sip of water. Furthermore, it accommodates high dose strengths which would be difficult for children to swallow. Although 3D printing of medicines was expected to revolutionize pediatric drug formulation, especially for niche drugs, Spritam® remains the only authorized 3D printed medicine to date. This limited adoption may be attributable to the relatively higher cost of 3D printing and lower throughput, compared to conventional tablets and capsules.

**Fig. 6:**
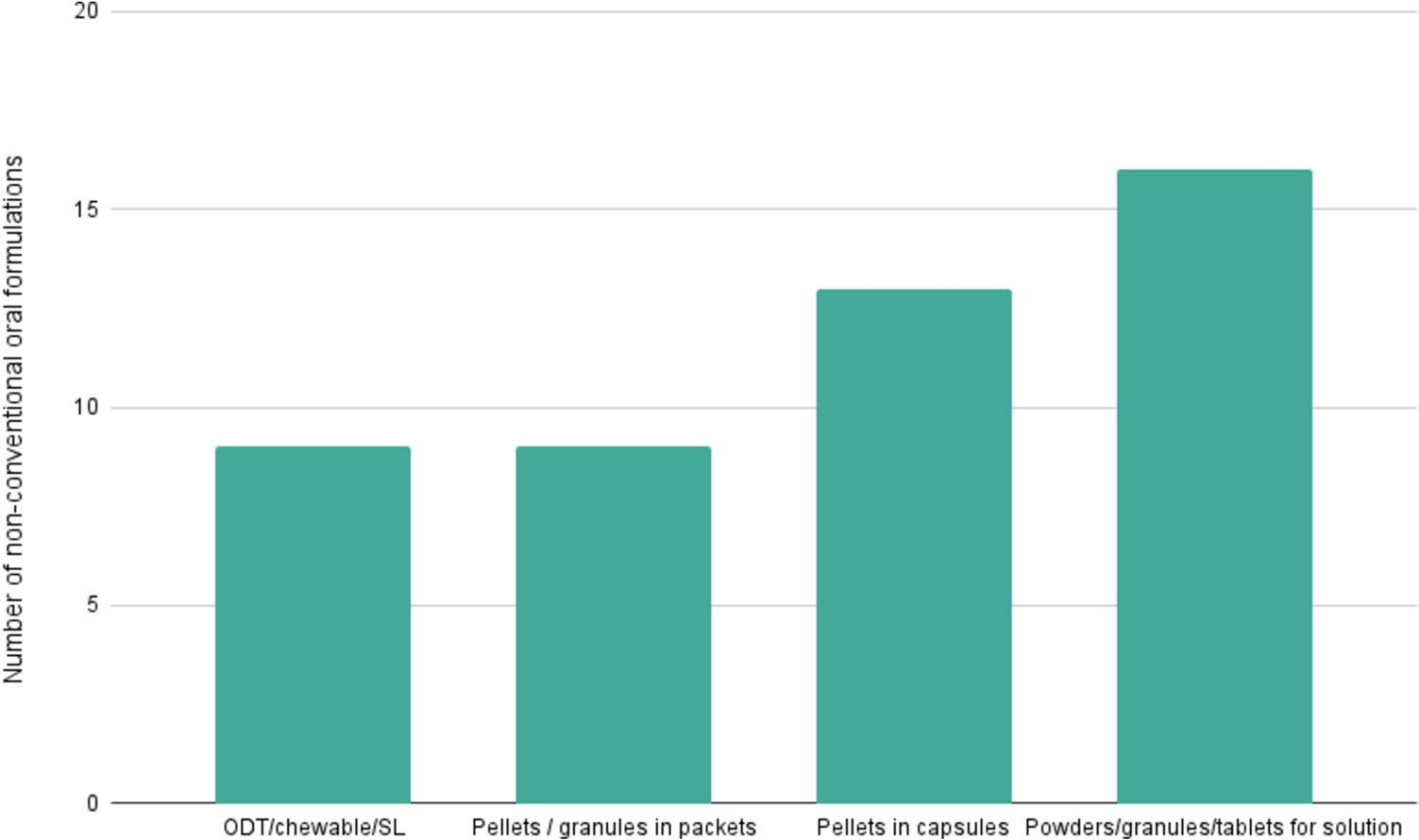
Distribution of non-conventional oral dosage forms authorized for pediatrics. Note. ODT: orally disintegrating tablets; SL: sublingual.

Three other authorized medicines for pediatrics are ODTs, including, Lamictal ODT® (lamotrigine 25, 50, 100, 200 mg) (GlaxoSmithkline, NC, USA), COTEMPLA XR-ODT® (methylphenidate 8.6, 17.3, 25.9 mg biphasic drug release ion-exchange resin formulation) (Neos Therapeutics, Inc. CO, USA) and Adzenys XR-ODT® (amphetamine 3.1, 6.3, 9.4, 12.5, 15.7, 18.8 mg) (Neos Therapeutics, Inc. CO, USA). Chewable tablet formulations include Dilantin Infatabs® (phenytoin 50 mg) (Pfizer, NY, USA), Vyvanse® (lisdexamfetamine dimesylate 10, 20, 30, 40, 50, 60 mg) (Takeda, Osaka, Japan) and Quillichew ER® (methylphenidate 20, 30, 40 mg biphasic drug release ion-exchange resin formulation) (Tris Pharma, NJ, USA). Among all ODTs and chewable tablet formulations authorized for pediatric use, only one—Isentress® (raltegravir chewable tablet for HIV)—is not indicated for the nervous system. One sublingual tablet formulation is authorized for pediatrics—Saphris® (asenapine maleate 2.5 mg) (AbbVie, IL, USA), indicated for bipolar disorder in children ages 10–17 years. Figure 6 illustrates the distribution of non-conventional dosage forms.

**Fig. 7:**
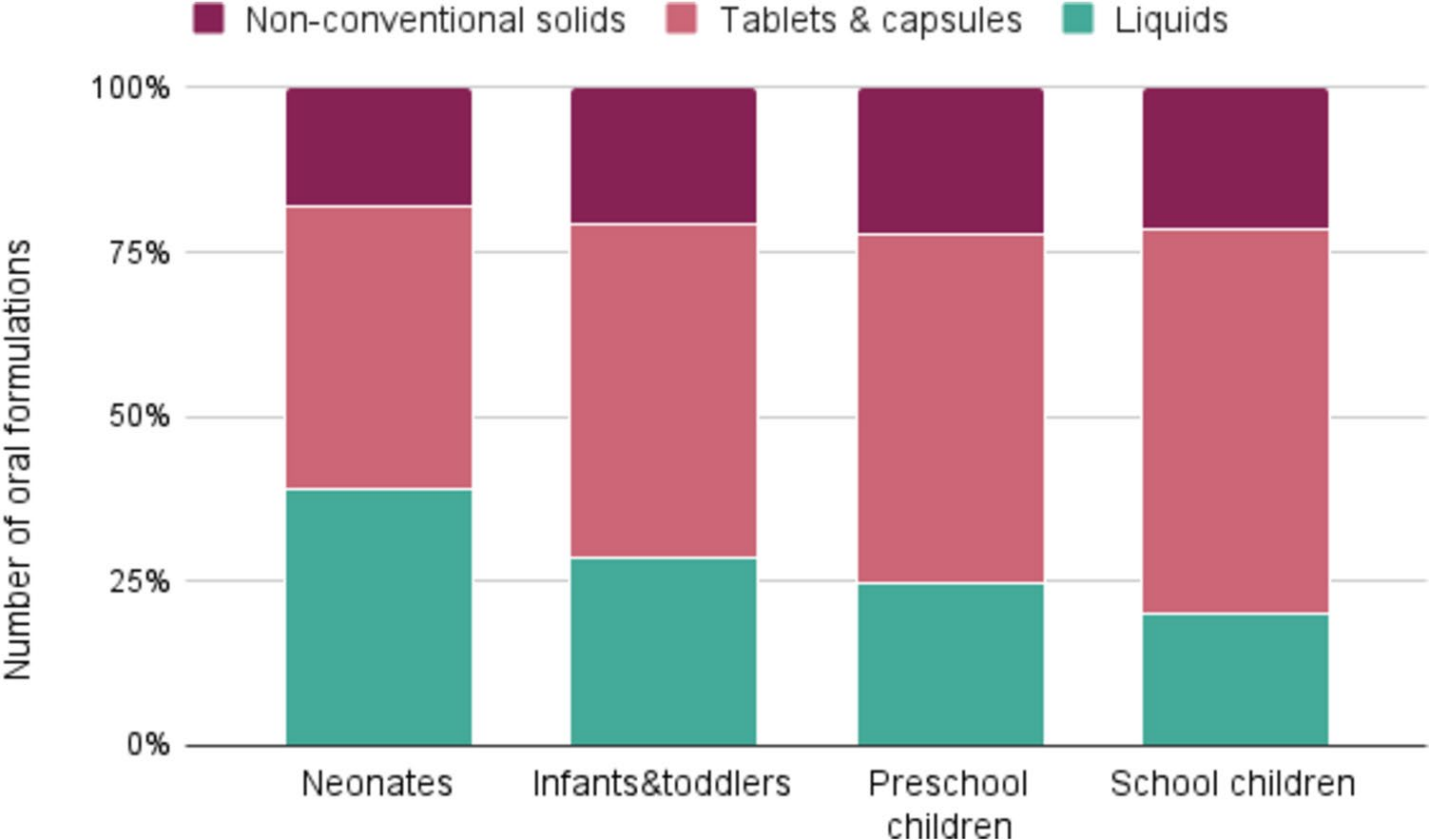
Distribution of oral authorized formulations across pediatric subgroups.

The proportion of liquid oral formulations authorized for pediatric use demonstrates a gradual decline with increasing age across the pediatric population (Fig. 7). Notably, although tablets and capsules comprise 43% and 51% of formulations for neonates, and infants and toddlers, respectively, it is pertinent to refer to the data presented in Table 2, which shows that many conventional solid dosage forms for this age group are available as alternative formulations that are more easily swallowed, although they contain excipients with known or potential safety concerns. Presence of excipients of concern in commercially available liquid formulations may provide a compelling argument for solid dosage form manipulation and compounding for these age groups [11]. Interestingly, the proportion of non-conventional solids remains approximately the same for each age group ranging from 17.9% in neonates to 22.1% for pre-school children.

### Excipients with Safety Concerns and Sweeteners in Authorized Pediatric Liquid Formulations

**Fig. 8:**
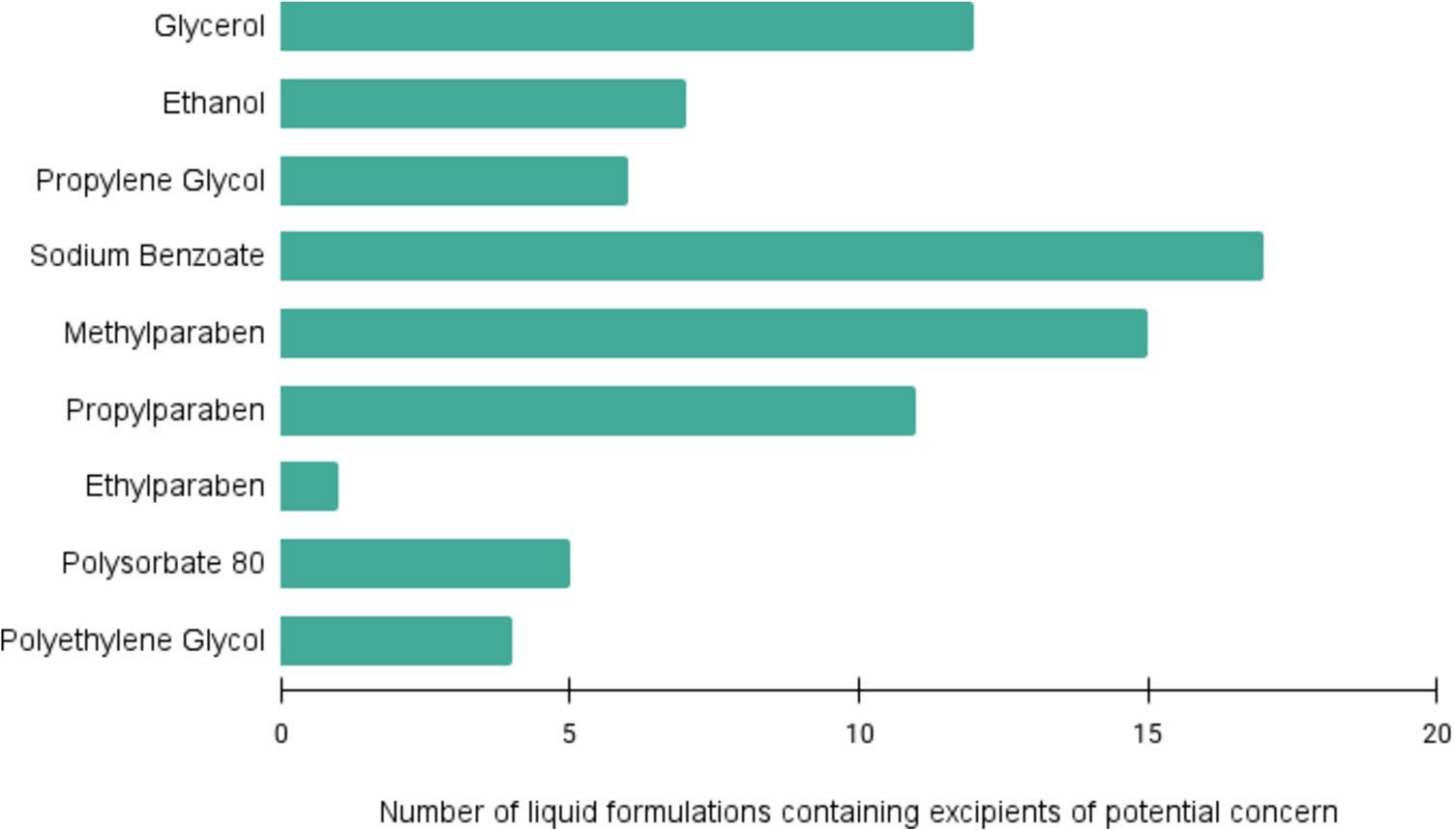
Number of oral liquid formulations authorized for pediatrics containing excipients with known or potential concerns.

Figure 8 presents an analysis of the prevalence of excipients associated with potential or known concerns in oral liquid formulations authorized for pediatric use. Identified excipients include ethanol, propylene glycol, glycerol, parabens, sodium benzoate, polysorbate 80, and polyethylene glycol [13, 37, 38]. The scope of this analysis was limited to liquid formulations, given that the quantities of such excipients in solid dosage forms are typically minimal, with ethanol and propylene glycol primarily serving as solvents or plasticizers in film coatings. Furthermore, it’s important to recognize that both the extent of exposure and the patient’s age are key determinants in excipients risk assessment. Excipients may have different pharmacokinetic and/or pharmacodynamic behavior in neonates and infants compared to school children [39, 40]. In this study, we only report the number of formulations containing excipients of potential concern; however, we do not assess the quantities of these excipients or whether their levels exceed acceptable limits. Quantitative composition data were not consistently available in the new drug applications (NDAs) or supplemental NDAs reviews, or patient information leaflets. The WHO PQPPAT considers only qualitative formulation composition and does not provide quantitative scoring guidance, an area that would benefit from further refinement. Nonetheless, documenting formulations that include excipients of concern remains important, given the potential for cumulative exposure in pediatric patients receiving multiple medications. For example, a recent study found that ethanol intake from medicinal products in children admitted to pediatric intensive care units could reach 2.18 mL per day [41].

Our study shows that ethanol, propylene glycol, and glycerol were the predominant cosolvents and wetting agents, present in 23 of the 43 pediatric liquid formulations assessed. Glycerol, the most frequently used excipient, serves as a density modifier, however carries the risk of osmotic diarrhea and at high doses,hypoglycemia, and metabolic (lactic) acidosis [42]. Ethanol and propylene glycol, both of which may exert adverse effects on hepatic function and the central nervous system, are subject to age-specific maximum limits as recommended by the EMA [43, 44]. Furthermore, alcohol poses a risk of pharmacodynamic interactions with drugs that potentiate central nervous system depression, including anxiolytics, antihistamines, hypnotics, and opioids [43, 45].

Parabens represent the most common preservatives in oral liquid formulations. Due to evidence of estrogenic activity and reduced spermatogenesis in animal models, EMA recommends a propylparaben limit of 2 mg/kg/day for pediatrics [46]. Sodium benzoate,the second most common preservative, is limited to 5 mg/kg/day [47, 48]. It is contraindicated in neonates due to its association with jaundice and metabolic acidosis and has also been linked to coughing and wheezing in children [37].

Polysorbate 80 is used as a surfactant in oral liquid preparations. It has been associated with hypersensitivity reactions, multiple organ failure and mortality in newborns receiving parenteral vitamin E [49]. Polyethylene glycol (PEG), used as a suspending agent and viscosity enhancer in oral liquid formulations, has been associated with nephrotoxicity,metabolic acidosis [51, 52], and perturbations of the gut microbiome,inducing a laxative effect at higher concentrations [53].

**Fig. 9:**
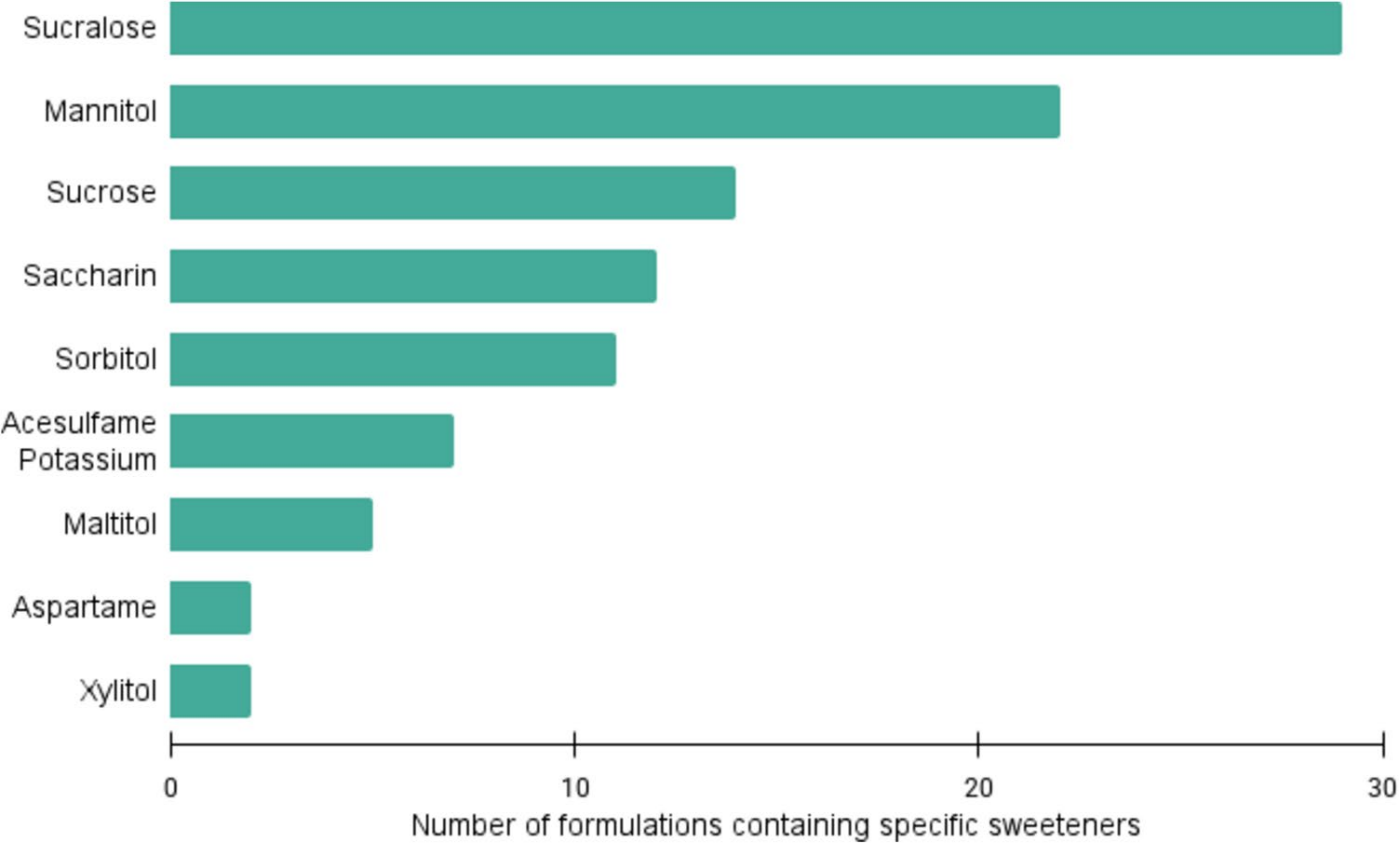
Prevalence of sweeteners in authorized oral liquid and non-conventional solid formulations authorized for pediatrics.

Figure 9 illustrates that sucralose is the most frequently utilized sweetener in authorized pediatric liquid and non-conventional solid formulations. Its selection is primarily due to its non-nutritive properties; unlike sucrose, sucralose poses no risk to diabetic patients and does not contribute to dental caries.

Mannitol ranks as the second most employed sweetener. It is favored in orodispersible and chewable tablets and is also incorporated into tablets for suspension, pellets, and granule-based formulations. Mannitol’s pleasant taste, negative enthalpy of dissolution, and non-hygroscopic nature make it well-suited for a wide range of pediatric dosage forms [51]. Sucrose is the third most prevalent sweetener, predominantly found in liquid preparations and in tablets or powders intended for suspension.

The STEP database (Safety and Toxicity of Excipients for Pediatrics) provides information on the safety of commonly used excipients; however, there is limited robust evidence available on acceptable exposure levels and maximum daily intake thresholds for children [50]. A pediatric excipient risk assessment framework has been proposed that incorporates key factors such as patient age, disease severity, dosing regimen, and route of administration to guide excipient selection in pediatric pharmaceutical development [54].

## Limitations

Our investigation was restricted to oral formulations authorized for pediatric use under FDA legislative frameworks: the Pediatric Rule, BPCA and PREA. A comparative evaluation involving pediatric-labeled formulations developed prior to the enactment of these Acts, or those used off-label, could provide deeper insights into the effectiveness of these regulatory measures in enhancing the age-appropriateness of pediatric formulations. Additionally, the scope of this study was limited to oral formulations and did not explore other routes of administration which also present acceptability challenges to pediatric patients. Nonethless, the study highlights the pitfalls of products that are assumed to be appropriate for children, therefore warranting the exploration of other routes of administration.

## Conclusions

Regulatory initiatives in the United States have contributed to improved access to efficacious and safe medicines for the pediatric population. Nonetheless, significant gaps persist in the availability of age-appropriate pharmaceutical formulations for children. In particular, authorized oral formulations for neonates, and infants and toddlers, remain limited compared to those available for older pediatric age groups. Moreover, only approximately half of the oral formulations approved for infants and toddlers, and preschool-aged children, meet age-appropriateness criteria. Tablets and capsules account for 58% of formulations indicated for pediatrics, despite tablet sizes often being unsuitable for younger children. Non-conventional solid dosage forms, including ODTs, chewables, pellets, and granules, constitute only approximately 20% of formulations for each age group, and therefore have not been fully leveraged in pediatric medicine. A considerable proportion of authorized liquid preparations contain excipients with safety concerns, including ethanol, propylene glycol and parabens. To address these challenges, robust regulatory frameworks are needed to guide the selection and development of optimal age-appropriate formulations tailored to specific pediatric subpopulations.

## Supplementary Information

Below is the link to the electronic supplementary material.

ESM 1 (XLSX 17.8 KB)

## Data Availability

Data supporting the findings of this study are available in the graphs of the article. Supplementary file lists the formulations evaluated in this study.

## Declarations

HRW reports current employment with Eli Lilly and Company and ownership of Lilly stock. This research was conceived and conducted entirely prior to the author’s employment at Eli Lilly and Company and is not affiliated with the company in any manner. Eli Lilly and Company had no involvement in the study design, data collection, data analysis, manuscript preparation, or decision to publish. The author’s contributions were made independently and outside the scope of employment.

## References

[1] Milne CP, Bruss JB. The economics of pediatric formulation development for off-patent drugs. Clin Ther. 2008;30(11):2133–45. 19108801 10.1016/j.clinthera.2008.11.019

[2] Petkova V, Georgieva D, Dimitrov M, Nikolova I. Off-label prescribing in pediatric population-literature review for 2012-2022. Pharmaceutics. 2023. 10.3390/pharmaceutics15122652. 38139994 10.3390/pharmaceutics15122652 PMC10747118

[3] European Medicines Agency. EMEA/126327/2004. Evidence of harm from off-label or unlicensed medicines in children 2004. Available from: https://www.ema.europa.eu/en/documents/other/evidence-harm-label-or-unlicensed-medicines-children_en.pdf. Accessed 11 May 2024

[4] Best Pharmaceuticals for Children Act, Pub. L. No. 107–109 2002. https://www.congress.gov/bill/107th-congress/senate-bill/1789/text. Accessed 11 May 2024.

[5] Pediatric Research Equity Act, Pub. L. No. 108–155 2003. https://www.congress.gov/bill/107th-congress/senate-bill/1789/text. Accessed 11 May 2024.

[6] Food and Drug Administration. Regulations requiring manufacturers to assess the safety and effectiveness of new drugs and biological products in pediatric patients. 1998. 21 CFR Parts 201, 312, 314, and 601 [Docket No. 97N–0165] RIN 0910–AB20. https://www.govinfo.gov/content/pkg/FR-1998-12-02/pdf/98-31902.pdf. Accessed 8 July 2024

[7] Regulation (EC) No 1901/2006 of the European Parliament and of the Council of 12 December 2006 on medicinal products for paediatric use 2006. https://eur-lex.europa.eu/legal-content/EN/TXT/?uri=CELEX%3A32006R1901. Accessed 11 May 2024.

[8] Commission to the European Parliament and the Council. State of Pediatric Medicines in the EU: 10 years of the EU Pediatric Regulation. 2017. https://health.ec.europa.eu/system/files/2017-11/2017_childrensmedicines_report_en_0.pdf. Accessed 11 May 2025

[9] Food and Drug Administration. Pediatric Labelling Changes 2025. https://www.fda.gov/science-research/pediatrics/pediatric-labeling-changes.

[10] Hwang TJ, Orenstein L, Kesselheim AS, Bourgeois FT. Completion rate and reporting of mandatory pediatric postmarketing studies under the US Pediatric Research Equity Act. JAMA Pediatr. 2019;173(1):68–74. 30452498 10.1001/jamapediatrics.2018.3416 PMC6583440

[11] Fadda HM, Weiler H, Carvalho M, Lee YZ, Dassouki H, AbuBlan R, et al. Pediatric oral extemporaneous preparations and practices: International Pharmaceutical Federation (FIP) global study. Eur J Pharm Biopharm. 2024;204: 114483. 39245358 10.1016/j.ejpb.2024.114483

[12] European Medicines Agency. Guideline on pharmaceutical development of medicines for pediatric use. EMA/CHMP/QWP/805880/2012 Rev. 2.2014. https://www.ema.europa.eu/en/documents/scientific-guideline/guideline-pharmaceutical-development-medicines-paediatric-use_en.pdf. Accessed 11 May 2024.

[13] Quijano Ruiz B, Desfontaine E, Arenas-Lopez S, Wang S. Pediatric formulation issues identified in paediatric investigation plans. Expert Rev Clin Pharmacol. 2014;7(1):25–30. 24308789 10.1586/17512433.2014.857600

[14] Orubu ESF, Duncan J, Tuleu C, Turner MA, Nunn A. WHO essential medicines for children 2011-2019: age-appropriateness of enteral formulations. Arch Dis Child. 2022;107(4):317–22. 34479858 10.1136/archdischild-2021-321831

[15] World Health Organization. WHO pediatric quality product profile assessment tool. 2024. https://www.who.int/publications/m/item/who-paediatric-quality-product-profile-assessment-tool. Accessed 11 May 2024.

[16] Food and Drug Administration. Guidance for Industry: Q8(R2) Pharmaceutical Development 2009. https://www.fda.gov/media/71535/download. Accessed 3 Aug 2025.

[17] Williams K, Thomson D, Seto I, Contopoulos-Ioannidis DG, Ioannidis JP, Curtis S, et al. Standard 6: age groups for pediatric trials. Pediatrics. 2012;129(Suppl 3):S153–60. 22661762 10.1542/peds.2012-0055I

[18] International Council for Harmonisation. Clinical Investigation of Medicinal Products in the Pediatric Population. E11 (R1). 2017. https://database.ich.org/sites/default/files/E11_R1_Addendum.pdf. Accessed 2 July 2024.

[19] World Health Organization. Weight-for-age. 2024. https://www.who.int/tools/child-growth-standards/standards/weight-for-age.

[20] Walsh J, Masini T, Huttner BD, Moja L, Penazzato M, Cappello B. Assessing the appropriateness of formulations on the WHO model list of essential medicines for children: development of a paediatric quality target product profile tool. Pharmaceutics. 2022. 10.3390/pharmaceutics14030473. 35335850 10.3390/pharmaceutics14030473 PMC8950931

[21] Klingmann V, Linderskamp H, Meissner T, Mayatepek E, Moeltner A, Breitkreutz J, et al. Acceptability of multiple uncoated minitablets in infants and toddlers: a randomized controlled trial. J Pediatr. 2018;201(202–7): e1. 10.1016/j.jpeds.2018.05.031 29960767

[22] Munch J, Meissner T, Mayatepek E, Wargenau M, Breitkreutz J, Bosse HM, et al. Acceptability of small-sized oblong tablets in comparison to syrup and mini-tablets in infants and toddlers: a randomized controlled trial. Eur J Pharm Biopharm. 2021;166:126–34. 34153451 10.1016/j.ejpb.2021.06.007

[23] El Edelbi R, Eksborg S, Lindemalm S. In situ coating makes it easier for children to swallow and tolerate tablets and capsules. Acta Paediatr. 2015;104(9):956–61. 25982837 10.1111/apa.13041 PMC4744733

[24] Van Hemelryck S, Van Landuyt E, Hufkens V, Vanveggel S. Assessment of swallowability and acceptability of scored darunavir/cobicistat/emtricitabine/tenofovir alafenamide (D/C/F/TAF) fixed-dose combination (FDC) tablets in HIV-1-infected children aged >/=6 to <12 years, using matching placebo tablets: a randomized study. Antivir Ther. 2024;29(2):13596535241248282. 38725258 10.1177/13596535241248282

[25] Rashed AN, Terry D, Fox A, Christiansen N, Tomlin S. Feasibility of developing children’s pill school within a UK hospital. Arch Dis Child. 2021;106(7):705–8. 33229414 10.1136/archdischild-2020-319154

[26] DailyMed: National Institute of Health, National Library of Medicines; https://dailymed.nlm.nih.gov/dailymed/index.cfm. Accessed 6 Oct 2025

[27] Lexi-Drugs. Lexicomp Online: Wolters Kluwer Health, Inc. http://online.lexi.com. Accessed 11 Oct 2025.

[28] World Health Organization. Anatomic Therapeutic Chemical (ATC) and Defined Daily Dose (DDD) Toolkit. 2024. https://www.who.int/tools/atc-ddd-toolkit. Accessed 11 Oct 2025.

[29] Abrams EJ, Simonds RJ, Modi S, Rivadeneira E, Vaz P, Kankasa C, et al. PEPFAR scale-up of pediatric HIV services: innovations, achievements, and challenges. J Acquir Immune Defic Syndr. 2012;60(Suppl 3):S105–12. 22797731 10.1097/QAI.0b013e31825cf4f5 PMC4941954

[30] U.S. Department of State. PEPFAR 3.0 Controlling the Epidemic: Delivering on the Promise of an AIDS-free Generation 2014. https://2021-2025.state.gov/wp-content/uploads/2019/08/PEPFAR-3.0-–-Controlling-the-Epidemic-Delivering-on-the-Promise-of-an-AIDS-free-Generation.pdf. Accessed 6 Oct 2025.

[31] Rei Bolislis W, Bejeuhr G, Benzaghou F, Corriol-Rohou S, Herrero-Martinez E, Hildebrand H, et al. Optimizing pediatric medicine developments in the European Union through pragmatic approaches. Clin Pharmacol Ther. 2021;110(4):871–9. 33411346 10.1002/cpt.2152 PMC8518420

[32] van Riet-Nales DA, de Jager KE, Schobben AF, Egberts TC, Rademaker CM. The availability and age-appropriateness of medicines authorized for children in The Netherlands. Br J Clin Pharmacol. 2011;72(3):465–73. 21477143 10.1111/j.1365-2125.2011.03982.x PMC3175516

[33] Pikkarainen PH, Raiha NC. Development of alcohol dehydrogenase activity in the human liver. Pediatr Res. 1967;1(3):165–8. 6080860 10.1203/00006450-196705000-00001

[34] Munch J, Sessler I, Bosse HM, Wargenau M, Dreesen JD, Loforese G, et al. Evaluating the acceptability, swallowability, and palatability of film-coated mini-tablet formulation in young children: results from an open-label, single-dose, cross-over study. Pharmaceutics. 2023. 10.3390/pharmaceutics15061729. 37376177 10.3390/pharmaceutics15061729 PMC10303492

[35] Lura V, Lura A, Breitkreutz J, Klingmann V. The revival of the mini-tablets: recent advancements, classifications and expectations for the future. Eur J Pharm Biopharm. 2025;210: 114655. 39922507 10.1016/j.ejpb.2025.114655

[36] Food and Drug Administration. Pediatric drug development: regulatory considerations — complying with the pediatric research equity act and qualifying for pediatric exclusivity under the best pharmaceuticals for children act 2023. https://www.fda.gov/regulatory-information/search-fda-guidance-documents/pediatric-drug-development-regulatory-considerations-complying-pediatric-research-equity-act-and. Accessed 23 Aug 2025

[37] Bobillot M, Delannoy V, Trouillard A, Kinowski JM, Sanchez-Ballester NM, Soulairol I. Potentially harmful excipients: state of the art for oral liquid forms used in neonatology and pediatrics units. Pharmaceutics. 2024. 10.3390/pharmaceutics16010119. 38258129 10.3390/pharmaceutics16010119 PMC10820197

[38] Belayneh A, Tadese E, Molla F. Safety and biopharmaceutical challenges of excipients in off-label pediatric formulations. Int J Gen Med. 2020;13:1051–66. 33204140 10.2147/IJGM.S280330 PMC7667588

[39] Tuleu C, Breitkreutz J. Educational paper: formulation-related issues in pediatric clinical pharmacology. Eur J Pediatr. 2013;172(6):717–20. 23111761 10.1007/s00431-012-1872-8

[40] Wang S. Formulations in paediatric investigation plans (PIPs): introduction to PIP quality section and regulatory framework. Int J Pharm. 2015;492(1–2):332–4. 25959119 10.1016/j.ijpharm.2015.05.016

[41] Isaac R, Khan I, Langley C. Ethanol intake of paediatric intensive care patients. Arch Dis Child. 2013;98(6):e1-e.

[42] Brothwell SL, Fitzsimons PE, Gerrard A, Schwahn BC, Stockdale C, Bowron A, et al. Glycerol intoxication syndrome in young children, following the consumption of slush ice drinks. Arch Dis Child. 2025;110(8):592–6. 40068898 10.1136/archdischild-2024-328109 PMC12320603

[43] European Medicines Agency. Information for the package leaflet regarding ethanol used as an excipient in medicinal products for human use 2019. https://www.ema.europa.eu/en/documents/scientific-guideline/information-package-leaflet-regarding-ethanol-used-excipient-medicinal-products-human-use_en.pdf. Accessed 2 Oct 2025

[44] European Medicines Agency. Propylene glycol used as an excipient. 2017. https://www.ema.europa.eu/en/documents/report/propylene-glycol-used-excipient-report-published-support-questions-and-answers-propylene-glycol-used-excipient-medicinal-products-human-use_en.pdf. Accessed 2 Oct 2025.

[45] Chan LN, Anderson GD. Pharmacokinetic and pharmacodynamic drug interactions with ethanol (alcohol). Clin Pharmacokinet. 2014;53(12):1115–36. 25267448 10.1007/s40262-014-0190-x

[46] European Medicines Agency. Reflection paper on the use of methyl- and propylparaben as excipients in human medicinal products for oral use 2015. https://www.ema.europa.eu/en/documents/scientific-guideline/reflection-paper-use-methyl-and-propylparaben-excipients-human-medicinal-products-oral-use_en.pdf. Accessed 2 Oct 2025.

[47] European Medicines Agency. Questions and answers on benzoic acid and benzoates used as excipients in medicinal products for human use. 2017. https://www.ema.europa.eu/en/documents/scientific-guideline/questions-and-answers-benzoic-acid-and-benzoates-used-excipients-medicinal-products-human-use_en.pdf. Accessed 2 Oct 2025.

[48] Petrus M, Bonaz S, Causse E, Rhabbour M, Moulie N, Netter JC, et al. Asthma and intolerance to benzoates. Arch Pediatr. 1996;3(10):984–7. 8952792 10.1016/0929-693x(96)81719-2

[49] Kriegel C, Festag M, Kishore RSK, Roethlisberger D, Schmitt G. Pediatric safety of polysorbates in drug formulations. Children Basel. 2019;7(1). 10.3390/children7010001. 10.3390/children7010001 PMC7022221 31877624

[50] European Paediatric Formulation Initiative. STEP database: Safety and Toxicity of Excipients for Paediatrics. https://step-db.ucl.ac.uk/eupfi/appDirectLink.do?appFlag=login. Accessed 26 Aug 2025.

[51] Pharmaceutical Excipients: Pharmaceutical Press. 2025. https://www.pharmaceuticalpress.com/products/pharmaceutical-excipients/. Accessed 17 Aug 2025.

[52] Rouaz K, Chiclana-Rodriguez B, Nardi-Ricart A, Sune-Pou M, Mercade-Frutos D, Sune-Negre JM, et al. Excipients in the paediatric population: a review. Pharmaceutics. 2021. 10.3390/pharmaceutics13030387. 33805830 10.3390/pharmaceutics13030387 PMC8000418

[53] Zhao M, Wang P, Sun X, Yang D, Zhang S, Meng X, et al. Detrimental impacts of pharmaceutical excipient PEG400 on gut microbiota and metabolome in healthy mice. Molecules. 2023. 10.3390/molecules28227562. 38005284 10.3390/molecules28227562 PMC10673170

[54] Agrawal A, Salunke S, Rumondor A, Thompson K, Caivano G, Walsh J, et al. Paediatric excipient risk assessment (PERA) tool and application for selecting appropriate excipients for paediatric dosage forms - Part 2. Eur J Pharm Biopharm. 2024;203:114447. 39122051 10.1016/j.ejpb.2024.114447

